# The impact of electronic device use on learning quality in young children: the mediating role of executive function and the moderating role of parental mediation

**DOI:** 10.3389/fpubh.2025.1609878

**Published:** 2025-08-13

**Authors:** Fangbing Qu, Shiqi He, Feifei Yu, Changwei Gu

**Affiliations:** ^1^College of Preschool Education, Capital Normal University, Beijing, China; ^2^Chongqing Dazu Teachers Training School, Chongqing, China; ^3^Suzhou Education Quality Monitoring Centre, Suzhou, China

**Keywords:** electronic device use, learning quality, executive function, parental mediation, preschool children, moderated mediation

## Abstract

The increasing use of screen-based electronic devices among young children raises concerns about their potential impact on learning quality. While moderate and guided digital media use may support cognitive engagement, excessive or unregulated use can impair executive function and reduce learning outcomes. Parental mediation may serve as a protective factor, but its specific moderating role remains unclear. This study examined the relationship between electronic device use and learning quality in preschool children, focusing on the indirect pathway through executive function and the moderating role of parental mediation. A total of 3,322 preschool children (aged 3–5 years) participated, with their parents/guardians completed the questionnaires including Electronic Device Use, Learning Quality Parent Evaluation Scale, Executive Function Behavior Rating Scale, and Parental Mediation Questionnaire. Results showed that electronic device use negatively predicted executive function, which in turn negatively predicted children’s learning quality. Parental mediation moderated the indirect pathway, with higher levels of mediation attenuating the negative impact. These findings suggest that while excessive device use risks cognitive development, active parental mediation can mitigate adverse effects, highlighting the need for family-centered interventions.

## 1 Introduction

The rapid proliferation of digital technologies in recent years has fundamentally transformed the daily lives of individuals, including young children. As electronic devices become increasingly integrated into family environments, preschool children are exposed to various digital content at an unprecedented rate. Electronic device use refers to children’s engagement with screen-based media (smartphones, tablets, TVs) operationalized through a multidimensional risk-exposure framework (1, 2). This includes four domains: access (device availability/location), frequency (duration/context of use), content (age-appropriateness/educational quality) co-viewing (interactive mediation practices). Although parents and educators often view electronic devices as educational tools that can support early learning and development, concerns have been raised about their potential negative impacts on children’s cognitive, emotional, and social development (3–5). In particular, the influence of electronic device use on young children’s learning quality has garnered substantial attention from both researchers and practitioners, as early learning quality is essential for later academic success and psychosocial adjustment (1, 2, 6, 7). Developmental outcomes in this study specifically denote neurocognitive, socioemotional, and academic trajectories impacted by digital exposure, including: executive function (EF in short, e.g., working memory, inhibitory control, cognitive flexibility), learning quality (as defined above), stress physiology (e.g., cortisol levels), and long-term academic skills (literacy/numeracy).

Learning quality is explicitly defined as a multidimensional construct encompassing five core competencies developed through children’s daily learning activities (8): curiosity and interest (e.g., exploratory questioning, seeking novelty), initiative (e.g., self-directed engagement, proactive problem-solving), persistence and attention (e.g., task-focused endurance despite challenges), imagination and creativity (e.g., generating novel ideas, symbolic play), reflection and explanation (e.g., articulating thought processes, evaluating outcomes). This construct develops through dynamic child-environment interactions: cognitive maturation (e.g., EF growth) enables sustained attention and flexible thinking (9). Caregiver scaffolding (e.g., guided exploration, responsive feedback) cultivates intrinsic motivation and strategy use (10). Activity design (e.g., play-based vs. structured tasks) shapes behavioral engagement (11). Key factors influencing its development include: Child characteristics (e.g., temperament, EF), which are strongly linked to early academic success (6); Parental practices (e.g., mediation style, learning support) that directly shape EF through co-regulatory interactions (12); and environmental inputs (e.g., resource availability, digital exposure). EF—particularly inhibitory control and working memory—serve as foundational cognitive pillars for learning behaviors such as task persistence and curiosity (9). Notably, warm, responsive parenting buffers EF against environmental stressors (13), yet few studies integrate digital exposure, EF, and parenting within a unified model. On one hand, interactive and educational media have been shown to facilitate cognitive engagement and skill development when used appropriately under adult supervision (14, 15). On the other hand, excessive or unregulated use of electronic devices has been associated with reduced attention spans, impaired executive function, and poorer social interactions, raising concerns about the potential detrimental effects on learning quality (16, 17). Beyond learning quality, excessive electronic device use has been linked to broader neurocognitive alterations. Neuroimaging studies indicate that high screen exposure correlates with reduced cortical thickness in frontal and temporal regions, which are critical for EF and language processing (1, 2). Such structural changes may underpin deficits in attention control and working memory (18). Electronic device use is also associated with physiological stress markers, including elevated cortisol levels and disrupted sleep architecture, which impair cognitive recovery and emotional regulation (19). Furthermore, prolonged sedentary behavior during device use often displaces physical activity, leading to poorer motor coordination and cardiovascular fitness (9, 20). These cascading effects may compromise long-term academic success; longitudinal studies report that excessive electronic device use in early childhood predicts lower literacy and numeracy skills in later schooling (21, 22).

Critically, executive function (EF), encompassing inhibition, emotion control, shifting, planning and organization, and working memory (23), plays a pivotal role in preschool children’s learning quality. EF’s centrality to early education is well-established: It mediates socioeconomic impacts on school readiness (24), predicts math and literacy outcomes (25), and is modifiable through parenting interventions (26). However, digital media research often overlooks EF’s role as a mediator between device use and learning (6), a gap our study directly addresses. Consequently, we position EF not merely as a correlate but as the mediating mechanism translating digital exposure into learning outcomes, consistent with evidence that EF mediates environmental effects on academic skills (27).

In particular, the role of parental mediation in moderating the relationship between electronic device use and children’s developmental outcomes remains underexplored. While some studies suggest that parental involvement can buffer the negative effects of screen time by guiding content selection and promoting interactive use (28), others indicate that inconsistent or permissive mediation practices may exacerbate the risks associated with high device use (29, 30). Therefore, it is critical to investigate not only the direct effects of electronic device use on young children’s learning quality but also the complex interplay between device use, EF development, and parental mediation.

Research on the effects of electronic device use on young children’s developmental outcomes has produced mixed findings. On one hand, some studies highlight the potential benefits of educational technology, particularly when used in moderation and guided by adults who scaffold children’s understanding through questioning, contextual reinforcement, and co-engagement. For instance, interactive media that incorporate educational content can enhance cognitive skills, such as problem-solving, memory retention, and language development when adults actively contextualize content and bridge digital experiences to real-world learning (31). Recent evidence confirms that digital applications specifically designed for early education support vocabulary acquisition and early literacy primarily when caregivers engage in dialogic interaction (e.g., explaining concepts, relating content to daily life) during and after use (14, 15). Moreover, digital storytelling and educational games have been shown to stimulate curiosity and foster cognitive flexibility, especially when adults facilitate reflective discussion or extend digital play into physical activities (32).

Despite these potential benefits, a growing body of literature points to the negative impacts of excessive or unregulated electronic device use on children’s cognitive and socio-emotional development. Numerous studies indicate that high screen time is associated with impairments in EF, including difficulties in inhibition control, working memory, and cognitive flexibility (1, 2). For example, a longitudinal study revealed that preschool children who spent more than 2 h per day on screen activities exhibited poorer performance on measures of attention and self-regulation compared to their peers with limited screen exposure (33). This aligns with other research suggesting that passive consumption of digital content, particularly without adult interaction, can lead to reduced attentional control and decreased social engagement (34).

Parental mediation plays a crucial role in shaping how electronic device use influences children’s development. Studies have categorized parental mediation strategies into three main types: active mediation (discussing content with children), restrictive mediation (setting limits on use), and co-use (engaging with children during device use) (10). Active mediation, in particular, has been associated with positive developmental outcomes, as parents who guide their children through digital interactions help enhance learning comprehension and critical thinking. In contrast, restrictive mediation, when overly strict or inconsistent, may lead to increased curiosity and covert device use, while co-use without meaningful engagement fails to mitigate the negative effects (28–30). However, despite the recognition of parental mediation as a moderating factor, there is limited empirical evidence specifically examining how different mediation strategies interact with electronic device use to influence learning quality in early childhood. This research aims to address these gaps by systematically examining the moderating role of parental mediation in the relationship between electronic device use and learning quality, with a particular focus on the mediating role of EF.

Despite growing awareness of the potential impacts of electronic device use on young children’s learning quality, existing research still faces several significant gaps. To fill these gaps, the present study proposes a moderated mediation model to investigate how EF mediates the relationship between electronic device use and young children’s learning quality, and how parental mediation practices moderate the indirect pathway (i.e., electronic device use → executive function → learning quality). Specifically, we hypothesize the following:

1. Electronic device use negatively predicts learning quality in young children.
2. Given EF’s role as a neurocognitive conduit for digital influences, it will mediate the relationship between electronic device use and learning quality.
3. Because parental mediation scaffolds cognitive processing during device use, it will moderate the indirect pathway (electronic device use → executive function → learning quality), attenuating negative effects.

## 2 Methods

### 2.1 Participants

This study employed a stratified random sampling method to recruit preschool children aged 3–5 years and their primary caregivers from diverse family backgrounds. Eligible participants met the following criteria: (a) children aged between 3 and 5 years currently enrolled in preschool; (b) primary caregivers fluent in Chinese and able to complete questionnaires; and (c) written informed consent provided by parents or guardians. Participants were excluded if: (a) the child had been diagnosed with a neurodevelopmental disorder (e.g., Autism Spectrum Disorder, Attention Deficit Hyperactivity Disorder); (b) questionnaires had more than 20% missing data; or (c) the primary respondent was a non-residential caregiver (e.g., grandparents as custodians without parental oversight).

Initially, 3,459 child-caregiver dyads participated in the survey. After applying the exclusion criteria, 137 responses were removed, resulting in a final analytic sample of 3,322 dyads (retention rate: 96.03%). Detailed demographic characteristics are provided in Table 1. The final sample included children from junior classes (3-year-olds, 29.2%), middle classes (4-year-olds, 32.1%), and senior classes (5-year-olds, 38.7%). Among these participants, 1,691 (50.9%) were boys, and 1,631 (49.1%) were girls, with an average age of 56.70 months (SD = 10.84). Approximately 58.6% of the children attended public kindergartens, while 41.4% attended private institutions. Questionnaires were predominantly completed by mothers (76.4%), followed by fathers (23.3%) and other guardians (0.4%). Parental education levels were distributed as follows: junior high school or below (5.5%), high school or vocational training (15.1%), college diploma (23.3%), bachelor’s degree (45.2%), and master’s degree or higher (10.8%). Informed consent was obtained from the participants, and the study was approved by the ethics committee of the College of Preschool Education, Capital Normal University.

**Table 1:** Descriptive statistics of electronic device use, parental mediation, executive function, and learning quality.

Variable	Min	Max	Mean (M)	Standard Deviation (SD)	Skewness (Sk)	Kurtosis (Kur)
Electronic Device Use	0	20	8.07	2.91	0.53	0.55
Parental Mediation	25	125	98.92	18.22	−0.54	0.03
Executive Function	15	45	38.27	5.01	−0.70	0.24
Learning Quality	6	123	77.16	19.28	0.18	−0.05

### 2.2 Measures

#### 2.2.1 Electronic device use questionnaire

Children’s engagement with screen-based electronic devices was measured using the ScreenQ questionnaire (1, 2), a validated 15-item (16-question) parent-report tool designed to assess adherence to American Academy of Pediatrics (AAP) recommendations. The measure evaluates four domains derived from AAP guidelines: Access: Availability and location of devices (Items 1–5). Frequency: Duration and context of daily use (Items 6–9). Content: Age-appropriateness and educational quality (Items 10–12). Co-viewing: Interactive mediation practices (Items 13–15, question 13 is divided into 13a (Co-view TV/videos) and 13b (Co-use games/apps), so there are 2 questions). Responses use binary (0/1), ordinal (0–2), or frequency-based scales, translated to ordinal scores consistent with AAP risk thresholds (e.g., >1 h/day = 2 points). Total scores range from 0 to 26, where higher scores indicate greater non-adherence to AAP guidelines (1, 2). For this study, we culturally adapted ScreenQ through forward-backward translation and pilot testing with Chinese parents, ensuring item clarity. Cronbach’s *α* was 0.74, confirming acceptable reliability in our sample.

#### 2.2.2 Learning quality parent evaluation scale

The Learning Quality Parent Evaluation Scale, developed by Cai (8), consists of 41 items rated on a 0–3 scale, where “always” is scored as 3, “often” as 2, “occasionally” as 1, and “never” as 0. The scale measures five dimensions: curiosity and interest (8 items), initiative (9 items), persistence and attention (9 items), imagination and creativity (9 items), and reflection and explanation (6 items), with higher total scores indicating better learning quality. The Cronbach’s alpha coefficient for this scale was 0.963, with a construct validity of 0.976, indicating high reliability and validity.

#### 2.2.3 Preschool children’s executive function behavior rating scale (parent version)

Developed by Gioia et al. (23), this scale assesses preschool children’s EF from the perspective of parents. The scale contains 15 items rated on a 1–3 scale: “completely consistent” scored as 1, “consistent” as 2, and “inconsistent” as 3. It includes five dimensions: inhibition (3 items), emotion control (3 items), shifting (3 items), planning and organization (3 items), and working memory (3 items). Higher scores indicate better EF. Scores from these dimensions were aggregated into a global EF score, reflecting EF as a holistic cognitive mechanism. The Cronbach’s alpha coefficient for this study was 0.838, and the construct validity was 0.815.

#### 2.2.4 Parental mediation of electronic device use questionnaire

Developed by Valcke et al. (35), this scale contains 25 items rated on a 1–5 scale, where “never” is scored as 1 and “always” as 5. It measures two dimensions: control (11 items, including regulation, prohibition, and usage rules) and warmth (14 items, including communication and support), with higher scores indicating more frequent parental mediation behaviors. The Cronbach’s alpha coefficient was 0.952, and the construct validity was 0.958, indicating high reliability and validity.

### 2.3 Data analysis

Data were processed using SPSS 26.0 and Mplus 8.3. To evaluate potential common method bias arising from parent-reported measures, we conducted Harman’s single-factor test via exploratory factor analysis (EFA) on all questionnaire items. Results indicated 17 factors with eigenvalues >1, with the largest factor accounting for 24.69% of variance [below the 40% threshold; Podsakoff et al. (36)], suggesting no severe common method bias. Descriptive statistics, correlation analysis, and structural equation modeling were used to examine the relationships among variables. The mediation effect was tested using the bootstrap method (5,000 resamples), and the moderation effect was tested using the latent moderated structural equation (LMS) method.

## 3 Results

### 3.1 Common method bias test

To examine whether common method bias exists, we employed the widely used Harman’s single-factor test. Exploratory factor analysis was conducted on all items. A total of 17 common factors with eigenvalues greater than 1 were identified. The first factor explained 24.69% of the total variance, which is below the critical value of 40%, indicating that common method bias is not significant in this study.

### 3.2 Descriptive statistics

The results of descriptive statistics for electronic device use, parental mediation, EF, and quality of learning are shown in Table 1. The mean value of electronic device use of the children in the study was 8.07, indicating that the children in the study were more compliant with the recommendations of the AAP, China’s “Recommendations for Exercise Guidance for Children 0–6 Years of Age at Home During the Epidemic of Novel Coronavirus Pneumonia (First Edition)” regarding the use of electronic devices. The mean value of parental intervention was 98.92, indicating that all levels of parental intervention were high. The mean value of young children’s EF was 38.27, indicating that all levels of young children’s EF were high. The mean value of Learning Quality for young children was 77.16, indicating that the level of Learning Quality for young children was high.

### 3.3 Correlation analysis

We conducted correlation analysis among the variables: electronic device use, parental mediation, EF, and learning quality. The results are presented in Table 2. The findings revealed that electronic device use was significantly negatively correlated with parental mediation (*r* = −0.32, *p* < 0.01), EF (*r* = −0.30, *p* < 0.01), and learning quality (*r* = −0.26, *p* < 0.01). In contrast, parental mediation was positively correlated with EF (*r* = 0.28, *p* < 0.01) and learning quality (*r* = 0.47, *p* < 0.01), and EF was positively correlated with learning quality (*r* = 0.46, *p* < 0.01). These results indicate that there are significant pairwise correlations among electronic device use, parental mediation, EF, and learning quality.

**Table 2:** Correlation analysis between electronic device use, parental mediation, executive function, and learning quality.

Variable	1	2	3	4
Electronic device use	1			
Parental mediation	−0.32**	1		
Executive function	−0.30**	0.28**	1	
Learning quality	−0.26**	0.47**	0.46**	1

^*^p < 0.05, ^**^p < 0.01, ^***^p < 0.001.

### 3.4 Model testing

We used Mplus 8.3 to test a moderated mediation model examining (a) whether EF mediates the relationship between electronic device use and learning quality, and (b) whether parental mediation moderates this indirect pathway. Three equations were estimated to operationalize this model:

1. Equation 1 (Direct Effect): Tests the direct path from electronic device use (X) to learning quality (Y): Y = cXY = cX
2. Equation 2 (Mediator Effect): Tests the path from electronic device use (X) to EF (M): M = aXM = aX
3. Equation 3 (Moderated Mediation): Tests the full model, including the moderated path from EF (M) to learning quality (Y) by parental mediation (W) and their interaction (M × W): Y = c′X + bM + dW + e(M × W)Y = c′X + bM + dW + e(M × W)

**Figure 1:**
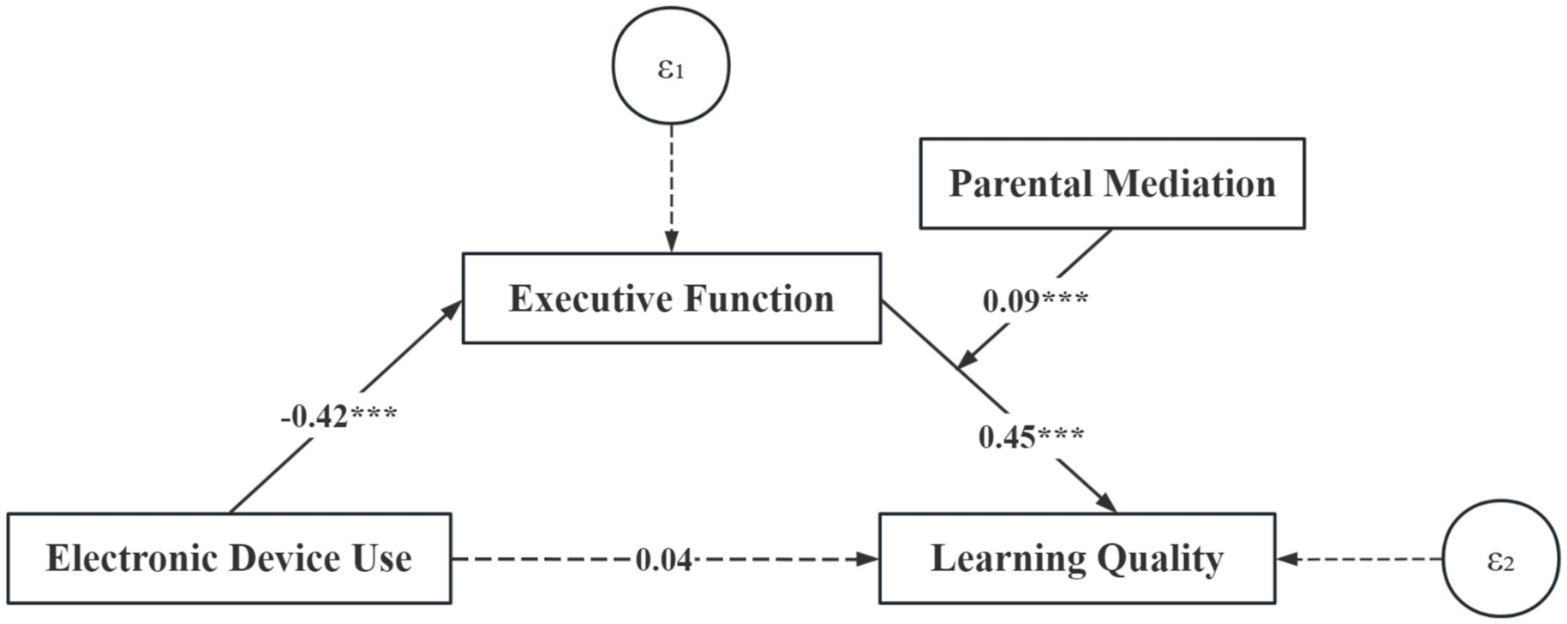
Moderated mediation model (all path coefficients are normalized path coefficients).

Model fit indices were acceptable: χ^2^/df = 27.56, CFI = 0.96, TLI = 0.94, RMSEA = 0.07, SRMR = 0.06. Results confirmed that EF mediated the relationship between electronic device use and learning quality, and parental mediation moderated the latter half of this pathway (Figure 1, Table 3).

**Table 3:** Conditional indirect effect test.

Predictor variable	Equation 1			Equation 2			Equation 3			
	Learning quality			Executive function			Learning quality			
	*β*	*t*	95%CI	*β*	*t*	95%CI	*β*	*t*	*df*	95%CI
Electronic device use (X)	−0.24	−11.22^***^	[−0.29, −0.20]	−0.42	−19.94^***^	[−0.46,-0.38]	0.04	1.50	3,316	[−0.01, 0.08]
Executive function (M)							0.45	23.68^***^	3,316	[0.41, 0.48]
Parental mediation (W)							0.42	25.36^***^	3,316	[0.39, 0.45]
Interaction (X × W)							0.09	5.83^***^	3,316	[0.06, 0.12]
*R* ^2^	0.06			0.18			0.42			

^*^p < 0.05, ^**^p < 0.01, ^***^p < 0.001.

### 3.5 Moderated mediation effect

**Figure 2:**
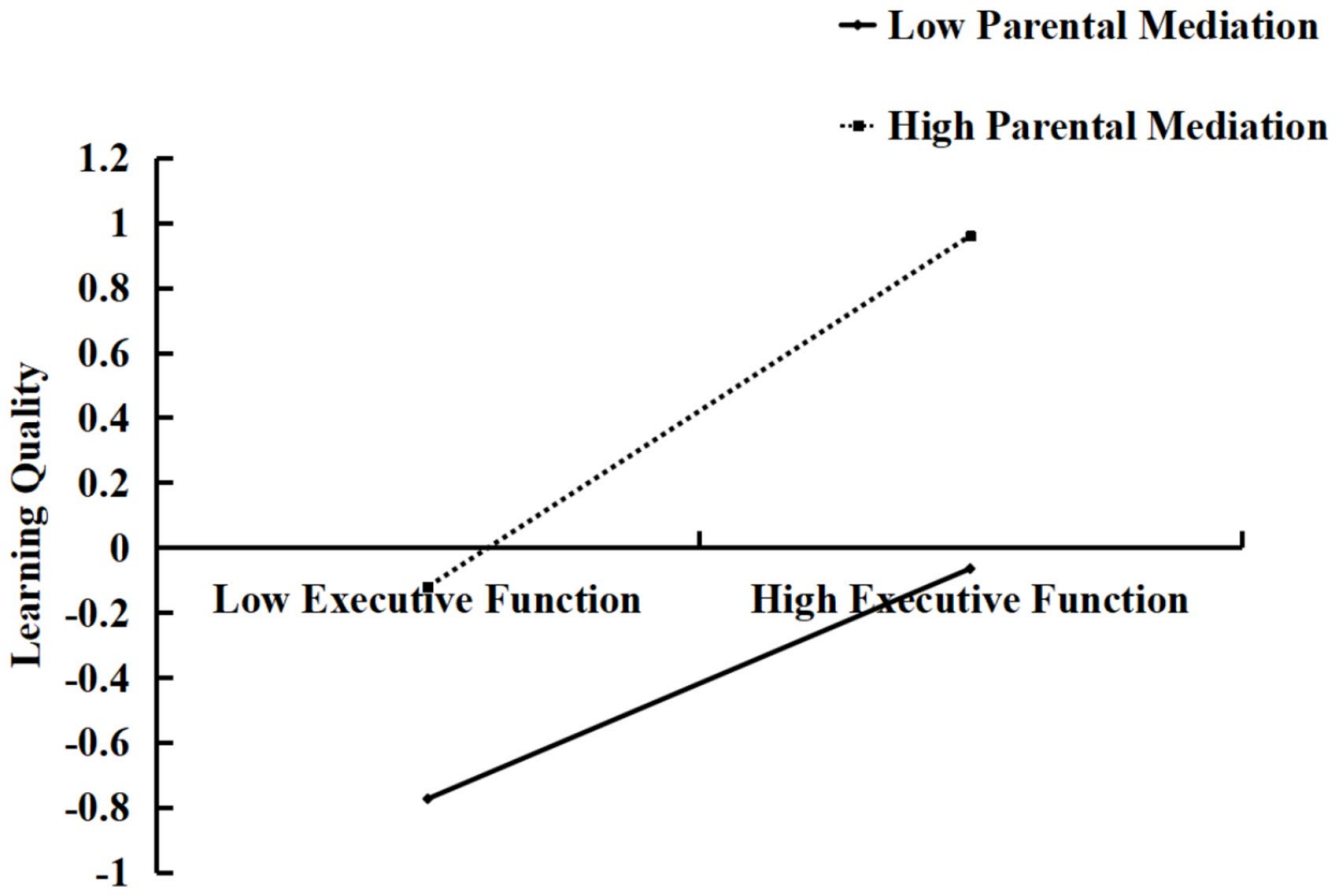
Moderating effect of parental mediation on the relationship between executive functioning and quality of learning.

To further investigate how parental mediation moderates the relationship between EF and learning quality, we categorized parental mediation into high (*M +* 1SD) and low (*M −* 1SD) groups. We then conducted a simple slope analysis. The results, as shown in Figure 2, indicated that when parental mediation was low (*M −* 1SD), the positive predictive effect of EF on learning quality was significant (*B_simple_* = 0.33, *t* = 14.72, *p* < 0.001). When parental mediation was high (*M* + 1SD), the positive predictive effect of EF on learning quality was significantly stronger (*B_simple_* = 0.50, *t* = 18.89, *p* < 0.001).

### 3.6 Conditional indirect effect analysis

As shown in Table 4, when parental mediation was low (*M* − 1SD), the indirect effect of electronic device use on learning quality through EF was −0.57, with a 95% Bootstrap confidence interval of [−0.60, −0.48], not including zero, indicating a significant mediation effect. When parental mediation was high (*M* + 1SD), the indirect effect of electronic device use on learning quality through EF was −0.33, with a 95% Bootstrap confidence interval of [−0.35, −0.28], also not including zero, indicating a significant difference in mediation effects between high and low parental mediation groups.

**Table 4:** Conditional indirect effect analysis.

Moderating variable	Indirect effect	95% confidence interval	
		Lower	Upper
Low parental mediation (−1SD)	−0.57	−0.60	−0.48
High parental mediation (+1SD)	−0.87	−0.90	−0.69
Difference	−0.30	−0.66	−0.21

^*^p < 0.05, ^**^p < 0.01, ^***^p < 0.001.

## 4 Discussion

This study aimed to examine the relationship between screen-based electronic device use (i.e., exposure to smartphones, tablets, TVs) and preschool children’s learning quality, specifically investigating the indirect effect of EF and the moderating role of parental mediation. We hypothesized that electronic device use would negatively predict learning quality, EF would mediate this relationship, and parental mediation would moderate the mediation effect by buffering these negative impacts. Contrary to our initial hypothesis regarding direct effects, we found no significant direct association between electronic device use and learning quality. Instead, our key finding was that electronic device use negatively predicted EF, which in turn negatively predicted children’s learning quality. This indicates that EF fully mediates the negative impact of electronic device use on learning quality. Additionally, parental mediation significantly moderated the indirect pathway through EF; higher levels of parental mediation attenuated the negative relationship between electronic device use and EF. These findings emphasize the central role of EF as a critical mediating mechanism and highlight parental mediation as a potential protective factor.

The most significant finding in this study was that EF fully mediated the relationship between electronic device use and learning quality. Specifically, increased electronic device use was associated with impaired executive function, which subsequently predicted lower learning quality in preschool-aged children. The nature of this relationship is rooted in neurodevelopmental vulnerability: Excessive screen-based device use disrupts prefrontal cortex maturation through reduced synaptic pruning and delayed myelination (1, 2), directly impairing EF like working memory and cognitive flexibility. These EF deficits manifest behaviorally as reduced attentional persistence (e.g., abandoning challenging tasks) and poor impulse control—core competencies underpinning learning quality (9). Mechanistically, screen exposure displaces cognitively enriching activities (e.g., symbolic play, social interaction) and overstimulates dopaminergic pathways, conditioning children to rapid reward cycles that erode sustained attention (21). Consequently, EF acts as the conduit through which fragmented attention and reduced cognitive control translate into poorer learning behaviors (e.g., diminished curiosity, reduced reflection). Consistent with studies showing that excessive screen time negatively impacts EF by reducing attention and cognitive control abilities (21, 37), our finding that electronic device use indirectly impairs learning quality via EF aligns with evidence linking screen time to neurocognitive inefficiencies [e.g., delayed neural responsivity during attention tasks; Law et al. (38)] and heightened stress reactivity (19). Reduced physical activity due to sedentary screen use may further compound these effects, as fitness levels are positively associated with EF development (39). Critically, early deficits in these domains predict poorer academic trajectories, including math and reading achievement (22).

Another important finding concerns the moderating role of parental mediation. Parental mediation buffers these effects through two synergistic mechanisms. Firstly, active mediation (e.g., co-viewing with explanatory dialogue) helps children encode screen content into schemas, reducing cognitive load and reinforcing neural connections for memory consolidation (14, 15).

Secondly, warmth-focused mediation (e.g., emotion-labeling during media use) supports children’s affective regulation, preventing stress-induced cortisol surges that impair prefrontal functioning (19).

Critically, restrictive mediation alone (e.g., time limits without engagement) fails to scaffold EF development, explaining mixed findings in prior literature (29). Our measure’s emphasis on active strategies clarifies why mediation robustly moderated the device use → EF pathway. These findings align with prior evidence showing that parental involvement—particularly active mediation involving guidance, discussion, and structured interaction around digital content—can buffer against potential developmental harms related to digital exposure (40). However, while our study revealed a clear moderating effect of parental mediation, previous research has shown inconsistent results regarding parental mediation’s protective effects. Some studies indicate that restrictive or inconsistent parental mediation can exacerbate negative outcomes by triggering frustration, anxiety, or covert usage among children (41). The robust protective effect observed in our study can be attributed to our measure’s emphasis on active, positive mediation behaviors (e.g., co-viewing and content-guided interaction), which recent research has demonstrated to have consistent protective effects on children’s cognitive development (28). Our study expands upon these findings by explicitly examining parental mediation as a moderating factor within a moderated mediation model, demonstrating its efficacy in supporting cognitive development even under conditions of high electronic device use.

Additionally, the absence of a direct effect of electronic device use on learning quality contradicts some previous studies. Previous studies frequently reported a direct negative association between excessive screen time and early learning outcomes, particularly academic readiness and cognitive skills (42, 43). Our finding of no direct effect contrasts with studies reporting direct negative associations between screen time and learning outcomes [e.g., Hu et al. (42) and Li et al. (43)]. This discrepancy may arise from methodological differences: prior work often examined bivariate relationships or omitted EF as a mediator. Our moderated mediation model explicitly accounts for this cognitive pathway, suggesting that electronic device use primarily impacts learning quality indirectly via EF. When EF is modeled as a mediator, the direct effect becomes nonsignificant, aligning with neurocognitive evidence that screen exposure affects learning through foundational cognitive processes (1, 2, 44). Recent literature underscores that EF is a core underlying mechanism that often mediates relationships between environmental factors and learning outcomes (27, 45). Furthermore, the present study found a complete mediation of the effect of electronic device use on learning quality through EF. Initially, we hypothesized only a partial mediation, expecting direct negative effects of electronic device use on children’s learning quality to persist. While we initially hypothesized partial mediation, EF fully mediated the effect—a finding that diverges from studies reporting both direct and indirect pathways [e.g., Likhitweerawong and Boonchooduang (45)]. The complete mediation by EF suggests device use impacts learning quality primarily through cognitive pathways rather than direct behavioral interference. This aligns with the cognitive bottleneck hypothesis (27): Screen-impaired EF creates bottlenecks in information processing, reducing resources for higher-order learning behaviors (e.g., curiosity, creativity). Without adequate EF, children cannot leverage environmental inputs—even educational content—effectively. Thus, parental mediation’s moderating role operates at this bottleneck: By preserving EF capacity (e.g., via guided co-use), it enables children to convert digital inputs into learning gains. Recent developmental literature emphasizes that EF such as inhibitory control, cognitive flexibility, and working memory are highly susceptible to environmental influences and critically underpin early academic achievement and classroom behavior (27). Therefore, our findings compellingly illustrate the centrality of EF in interpreting digital media impacts, suggesting that any adverse effects of device use are primarily cognitive rather than directly educational.

Additionally, the strength and consistency of parental mediation’s moderating effect were stronger than anticipated. While we initially recognized parental mediation as a potential protective factor, its robust role in buffering the negative influence of electronic devices on EF was noteworthy. The robust buffering effect of parental mediation contrasts with studies reporting mixed or weak protective effects [e.g., Elias and Sulkin (41)]. This discrepancy may reflect measurement differences: our Parental Mediation Questionnaire (35) emphasizes active, co-engaged practices (e.g., discussion, guided interaction), whereas other studies focused on restrictive mediation, which can provoke reactance (29). Importantly, our results align with emerging evidence that active (not restrictive) mediation consistently mitigates cognitive risks (28), suggesting that mediation quality—not merely its presence—determines protective benefits. Our results align well with these findings, suggesting that parental mediation might be essential to preserving EF amid high electronic media exposure.

Finally, several limitations warrant acknowledgment. First, despite our large sample size (*N* = 3,322), participants were drawn exclusively from urban Chinese preschools, limiting socioeconomic and cultural diversity. Generalizability to rural populations, clinical subgroups (e.g., children with ADHD), or Western contexts requires validation.

Second, while the ScreenQ questionnaire aggregates device types (TVs, tablets, smartphones) and content purposes (educational/entertainment), we acknowledge this treats heterogeneous exposures uniformly. Future research should disaggregate these dimensions to examine differential impacts (e.g., passive TV viewing vs. interactive tablet use; educational apps vs. entertainment videos).

Third, the cross-sectional design precludes causal inferences about the relationships between electronic device use, EF, and learning quality. Longitudinal or experimental studies are needed to establish temporal precedence and rule out bidirectional effects (e.g., whether poor EF drives increased device use).

Fourth, while we controlled for key demographic variables (age, gender), unmeasured confounders—such as genetic predispositions, family stress levels, or school quality—may influence observed associations. Future research should incorporate multi-informant assessments (e.g., teacher reports of learning quality) and objective measures of device use (e.g., digital tracking) to reduce mono-method bias. Most critically, our exclusive reliance on parent-reported measures introduces significant limitations. While validated instruments were used, this approach risks response biases (e.g., social desirability in underreporting device use, subjective interpretations of EF). Direct behavioral assessments (e.g., NIH Toolbox, DCCS tasks) would provide more objective metrics but were precluded by our large-scale design (*N* = 3,322) and pandemic restrictions prohibiting researcher-child contact. Future studies should combine multi-informant reports with direct cognitive testing.

Notwithstanding these constraints, our moderated mediation model advances understanding of how parental mediation buffers cognitive risks in early digital exposure. Future work should employ longitudinal designs across diverse cultural contexts to verify these pathways and explore nuanced factors like content type (educational vs. entertainment) and co-use quality.

Despite these limitations, our findings have significant practical and theoretical implications. The identified full mediation role of EF indicates that structured interventions targeting preschool children’s cognitive skills—such as EF-integrated curricula involving memory games, inhibitory control tasks, and cognitive flexibility exercises—could effectively buffer the negative effects of electronic device use (27). Educators should model active mediation strategies during classroom screen activities by prompting open-ended discussions and connecting digital content to real-life contexts. Additionally, our results highlight parental mediation as a robust moderator, suggesting family-based interventions (e.g., workshops on active mediation techniques and promoting movement-based activity displacement) could further enhance EF and mitigate sedentary behaviors (46). Collaborative educator-parent efforts must emphasize consistency in mediation styles to prevent conflicting approaches from undermining these benefits (29).

## Data Availability

The raw data supporting the conclusions of this article will be made available by the authors, without undue reservation.

## References

[1] Hutton JS Dudley J Horowitz-Kraus T DeWitt T Holland SK. Associations between screen-based media use and brain white matter integrity in preschool-aged children. JAMA Pediatr. (2020) 174:e193869–1082. doi: 10.1001/jamapediatrics.2019.3869, PMID: 31682712 PMC6830442

[2] Hutton JS Huang G Sahay RD DeWitt T Ittenbach RF. A novel, composite measure of screen-based media use in young children (screen Q) and associations with parenting practices and cognitive abilities. JAMA Pediatr. (2020) 87:e203501. doi: 10.1038/s41390-020-0765-1, PMID: 32050256

[3] Gottschalk F. Impacts of technology use on children: exploring literature on the brain, cognition and well-being Oecd Education Working Paper no. 195. Organ Econ Co-Operation Dev 2019. (2019). Available online: www.oecd.org/edu/workingpapers (Accessed on 20 February 2025).

[4] Romeo RR Leonard JA Robinson ST West MR Mackey AP Rowe ML. Beyond the 30-million-word gap: Children’s conversational exposure is associated with language-related brain function. Psychol Sci. (2018) 29:700–10. doi: 10.1177/0956797617742725 29442613 PMC5945324

[5] Valkenburg PM Meier A Beyens I. Social media use and its impact on adolescent mental health: an umbrella review of the evidence. Curr Opin Psychol. (2022) 44:58–68. doi: 10.1016/j.copsyc.2021.08.017 34563980

[6] Huber B Yeates M Meyer D Fleckhammer L Kaufman J. The effects of screen media content on young children’s executive functioning. J Exp Child Psychol. (2018) 170:72–85. doi: 10.1016/j.jecp.2018.01.006, PMID: 29448235

[7] Supanitayanon S Trairatvorakul P Chonchaiya W. Screen media exposure in the first 2 years of life and preschool cognitive development: a longitudinal study. Pediatr Res. (2020) 88:894–902. doi: 10.1038/s41390-020-0831-8, PMID: 32170192

[8] Cai XX. Development and preliminary application of learning quality assessment tools for senior kindergarten children. [Master’s Thesis]. Xi’an, China: Shaanxi Normal University (2015).

[9] Diamond A Ling DS. Review of the evidence on, and fundamental questions about, efforts to improve executive functions, including working memory. In: Novick JM, ed. Cognitive and Working Memory Training: Perspectives from Psychology, Neuroscience, and Human Development. Oxford University Press; (2020) 143–431. doi: 10.1093/oso/9780199974467.003.0008

[10] Wright MF. The moderating effect of parental mediation in the longitudinal associations among cyberbullying, depression, and self-harm among Chinese and American adolescents. Front Psychol. (2024) 15:1459249. doi: 10.3389/fpsyg.2024.1459249 39737243 PMC11683589

[11] Bronfenbrenner U. The ecology of human development: Experiments by nature and design, vol. 352. Cambridge, Massachusetts, U.S.: Harvard university press (1979).

[12] Meeus A Eggermont S Beullens K. Constantly connected: the role of parental mediation styles and self-regulation in pre- and early adolescents’ problematic mobile device use. Hum Commun Res. (2018) 45:119–47. doi: 10.1093/hcr/hqy015

[13] Brown SM Schauer LH Harwich-Reiss E Dmitrieva J Miles E Watamura SI. Parental buffering in the context of poverty: positive parenting behaviors differentiate young children’s stress reactivity profiles. Dev Psychopathol. (2020) 32:1778–87. doi: 10.1017/S0954579420001224, PMID: 33427174 PMC9118882

[14] Medawar J Tabullo ÁJ Gago-Galvagno LG. Early language outcomes in Argentinean toddlers: associations with home literacy, screen exposure and joint media engagement. Br J Dev Psychol. (2023) 41:13–30. doi: 10.1111/bjdp.12429 35973831

[15] Burns T Gottschalk F, eds. Education in the Digital Age: Healthy and Happy Children. OECD Publishing (2020). doi: 10.1787/1209166a-en

[16] Bal M Aydemir AGK Cengiz GŞT Altındağ A. Examining the relationship between language development, executive function, and screen time: a systematic review. PLoS One. (2024) 19:e0314540. doi: 10.1371/journal.pone.0314540, PMID: 39724067 PMC11670964

[17] Adelantado-Rena M Moliner-Urdiales D Cavero-Redondo I Beltran-Valls MR Martinez-Vizcaino V Álvarez-Bueno C. Association between screen media use and academic performance among children and adolescents: a systematic review and meta-analysis. JAMA Pediatr. (2019) 173:1058–67. doi: 10.1001/jamapediatrics.2019.3176 31545344 PMC6764013

[18] Salahub C Lockhart HA Dube B Al-Aidroos N Emrich SM. Electrophysiological correlates of the flexible allocation of visual working memory resources. Sci Rep. (2019) 9:19428. doi: 10.1038/s41598-019-55948-4, PMID: 31857657 PMC6923388

[19] McDaniel BT Radesky JS. Technoference: parent distraction with technology and associations with child behavior problems. Child Dev. (2018) 89:100–109. doi: 10.1111/cdev.12822 28493400 PMC5681450

[20] Mollborn S Limburg A Pace J Fomby P. Family socioeconomic status and children’s screen time: implications for learning and development. J Marriage Fam. (2022) 84:1129–51. doi: 10.1111/jomf.12834 36211640 PMC9541918

[21] Madigan S Browne D Racine N Mori C Tough S. Association between screen time and children’s performance on a developmental screening test. JAMA Pediatr. (2019) 173:244–50. doi: 10.1001/jamapediatrics.2018.5056, PMID: 30688984 PMC6439882

[22] Mundy LK Canterford L Hoq M Olds T Moreno-Betancur M Sawyer S. Electronic media use and academic performance in late childhood: a longitudinal study. PLoS ONE. (2020) 15:e0237907. doi: 10.1371/journal.pone.0237908 32877427 PMC7467319

[23] Gioia GA Isquith PK Guy SC Kenworthy L. Test review: behavior rating inventory of executive function. Child Neuropsychol. (2000) 6:235–8. doi: 10.1076/chin.6.3.235.3152, PMID: 11419452

[24] Kao K Nayak S Doan SN Tarullo AR. Relations between parent EF and child EF: The role of socioeconomic status and parenting on executive functioning in early childhood. Transl Issues Psychol Sci. (2018) 4:122–37. doi: 10.1037/tps0000154

[25] Gözüm AC Kandir A. Digital games preschoolers play: parental mediation and examination of educational content. Educ Inf Technol. (2021) 26:3293–326. doi: 10.1007/s10639-020-10382-2

[26] Chen C Chen S. The impact of electronic device use on learning quality in young children: the mediating role of executive function and the moderating role of parental mediation. Adv Psychol. (2024) 14:141–46. doi: 10.12677/AP.2024.141020 PMC12380748 40880931

[27] Emslander V Scherer R. The relation between executive functions and math intelligence in preschool children: a systematic review and meta-analysis. Psychol Bull. (2022) 148:337–69. doi: 10.1037/bul0000369, PMID: 40658541

[28] Cao S Dong C Li H. Parental beliefs and mediation co-mediate the SES effect on chinese preschoolers’ early digital literacy: a chain-mediation model. Educ Inf Technol. (2024) 29:12093–114. doi: 10.1007/s10639-023-12300-8

[29] Çaylan N Yalçın SS Erat Nergiz M Yıldız D Oflu A Tezol Ö. Associations between parenting styles and excessive screen usage in preschool children. Turk Arch Pediatr. (2021) 56:261–6. doi: 10.14744/TurkPediatriArs.2020.43765, PMID: 34104919 PMC8152648

[30] Nagata JM Paul A Yen F Smith-Russack Z Shao IY Al-shoaibi AAA. Associations between media parenting practices and early adolescent screen use. Pediatr Res. (2025) 97:403–410. doi: 10.1038/s41390-024-03243-y 38834780 PMC11626836

[31] Dore RA Logan J Lin T-J Purtell KM Justice L. Characteristics of children’s media use and gains in language and literacy skills. Front Psychol. (2020) 11:2224. doi: 10.3389/fpsyg.2020.02224 33013579 PMC7509086

[32] Antrilli NK Wang S. Toddlers on touchscreens: immediate effects of gaming and physical activity on cognitive flexibility of 2.5-year-olds in the US. J Child Media. (2018) 12:496–513. doi: 10.1080/17482798.2018.1486332

[33] Radesky JS Kaciroti N Weeks HM. Longitudinal associations between use of Mobile devices for calming and emotional reactivity and executive functioning in children aged 3 to 5 years. JAMA Pediatr. (2023) 177:62–70. doi: 10.1001/jamapediatrics.2022.4793, PMID: 36508199 PMC9857453

[34] Zhao J Zhang Y Jiang F Ip P Ho FKW Zhang Y. Excessive screen time and psychosocial well-being: the mediating role of body mass index, sleep duration, and parent-child interaction. J Pediatr. (2018) 202:157–62. doi: 10.1016/j.jpeds.2018.06.029 30100232

[35] Valcke M Bonte S De Wever B Rots I. Internet parenting styles and the impact on internet use of primary school children. Comput Educ. (2010) 55:454–64. doi: 10.1016/j.compedu.2010.02.009

[36] Podsakoff PM MacKenzie SB Lee JY Podsakoff NP. Common method biases in behavioral research: a critical review of the literature and recommended remedies. J Appl Psychol. (2003) 88:879–903. doi: 10.1037/0021-9010.88.5.879, PMID: 14516251

[37] Walsh J Barnes J Tremblay M Chaput J-P. Associations between duration and type of electronic screen use and cognition in US children. Comput Hum Behav. (2020) 108:106312. doi: 10.1016/j.chb.2020.106312

[38] Law EC Han MX Lai Z Lim S Ong ZY Ng V. Associations between infant screen use, electroencephalography markers, and cognitive outcomes. JAMA Pediatr. (2023) 177:311–8. doi: 10.1001/jamapediatrics.2022.5674, PMID: 36716016 PMC9887532

[39] Valkenborghs SR Noetel M Hillman CH Nilsson M Smith JJ Ortega FB. The impact of physical activity on brain structure and function in youth: a systematic review. Pediatrics. (2019) 144:e20184032. doi: 10.1542/peds.2018-4032, PMID: 31554668

[40] Domoff SE Harrison K Gearhardt AN Gentile DA Lumeng JC Miller AL. Development and validation of the problematic media use measure: a parent report measure of screen media “addiction” in children. Psychol Pop Media Cult. (2019) 8:2–11. doi: 10.1037/ppm0000163, PMID: 30873299 PMC6411079

[41] Elias N Sulkin I. Screen-assisted parenting: the relationship between toddlers’ screen time and parents’ use of media as a parenting tool. J Fam Issues. (2019) 40:2801–2821. doi: 10.1177/0192513X19864983

[42] Hu BY Johnson GK Teo T Wu Z. Relationship between screen time and Chinese children’s cognitive and social development. J Res Child Educ. (2020) 34:183–207. doi: 10.1080/02568543.2019.1702600

[43] Li C Wang Y Zhang L Li X. The relationships between screen use and health indicators among infants, toddlers, and preschoolers: a meta-analysis and systematic review. Int J Environ Res Public Health. (2020) 17:7324. doi: 10.3390/ijerph17197324 33036443 PMC7579161

[44] McHarg G Ribner AD Devine RT Hughes C. Screen time and executive function in toddlerhood: a longitudinal study. Front Psychol. (2020) 11:570392. doi: 10.3389/fpsyg.2020.570392, PMID: 33192857 PMC7643631

[45] Likhitweerawong N Boonchooduang N. Executive dysfunction as a possible mediator for the association between excessive screen time and problematic behaviors in preschoolers. PLoS One. (2024) 19:e0298189. doi: 10.1371/journal.pone.0298189, PMID: 38574024 PMC10994291

[46] Valkenborghs SR Dent PC Stillman CM. The intergenerational effects of parental physical activity on offspring brain and neurocognition in humans: a scoping review. Neurosci Biobehav Rev. (2022) 143:104953. doi: 10.1016/j.neubiorev.2022.104953 36356681

